# Luminescence dating of glaciofluvial deposits linked to the penultimate glaciation in the Eastern Alps

**DOI:** 10.1016/j.quaint.2014.10.013

**Published:** 2015-01-30

**Authors:** Lukas Bickel, Christopher Lüthgens, Johanna Lomax, Markus Fiebig

**Affiliations:** a Institute of Applied Geology, Department of Civil Engineering and Natural Hazards, University of Natural Resources and Life Sciences, Peter Jordan-Straße 70, 1190 Vienna, Austria; b Department of Geography, Justus-Liebig-University Giessen, Senckenbergstraße 1, 35390 Giessen, Germany

**Keywords:** Penultimate glaciation, Deglaciation, Luminescence dating, Eastern Alps, Glaciofluvial deposits

## Abstract

During the penultimate glaciation vast areas of the Alps were glaciated, with piedmont glaciers protruding into the foreland. In the easternmost part of the northward draining valleys of the Alps, the glaciers did not reach the foreland, but formed valley glaciers confined by the mountainous terrain. This also applies to the Ybbs valley, where samples for luminescence dating out of glaciofluvial gravel accumulations were taken at three locations along the present day river course. In a highly dynamic depositional environment, such as a glacier-fed river system, incomplete resetting of the luminescence signal is possible, in particular when transport distances are short. In such cases, quartz usually is the preferred mineral over feldspar, especially if dose rates are low and may theoretically allow obtaining quartz ages beyond 150 ka. Because previous research has shown, and as corroborated within this study, quartz from the research area exhibits analytical problems in the high age range. Therefore luminescence properties of coarse grain (100–200 μm) quartz and in addition K-rich feldspar were investigated with the aim to reconstruct the chronology of the glacial processes within the Ybbs catchment area. Issues of incomplete bleaching were pIRIR225 encountered and addressed by comparing quartz OSL, fading corrected K feldspar IR50 and pIRIR225 to identify reliable ages. Depositional ages based on quartz OSL and feldspar pIRIR225 signals revealed deposition of ice marginal kame terraces and glaciofluvial foreland terraces during late to middle MIS 6. In combination with results from previous studies, we could reconstruct the valley evolution during the Riss glaciation. Newly gained luminescence ages of the deglaciation in the easternmost Alps coincide with OSL dated deglaciation events in the Western Alps, indicating that climatic change along the north side of the Alps happened simultaneously.

## 1 Introduction

The Alpine region and its foreland played a major role in the investigation of Quaternary glacial and paleo-climatic processes since the beginning of the 19th century (Agassiz, 1841). At the beginning of the 20th century, Penck and Brückner (1909) developed the model of the “glacial series” in the German part of the northern Alpine Foreland (NAF), a concept that describes the genetic connection of basal till, terminal moraines and adjoining outwash gravels. From the vertical succession of four gravel terrace levels, they developed a morphostratigraphic model which attributes a glacial period to each of these geological units (old to young: Günz, Mindel, Riss, Würm). Based on three elevated, morphologically distinguishable gravel levels in the German NAF, the quadriglacial system was amended by three additional glacials (Biber, Donau, Haslach) by Eberl (1930), Schaefer (1953), and Schreiner and Haag (1982). This morphostratigraphic model is still used in some alpine areas, but has been fine-tuned and amended since then, especially in terms of the chronostratigraphic position of the deposits. However, clear genetic relations in terms of the glacial series often can be ambiguous due to the complete lack of sedimentary remains and the often only poor preservation, especially those of the oldest glacials which underwent several cycles of severe geomorphological changes during subsequent glaciations and interglacials.

**Fig. 1:**
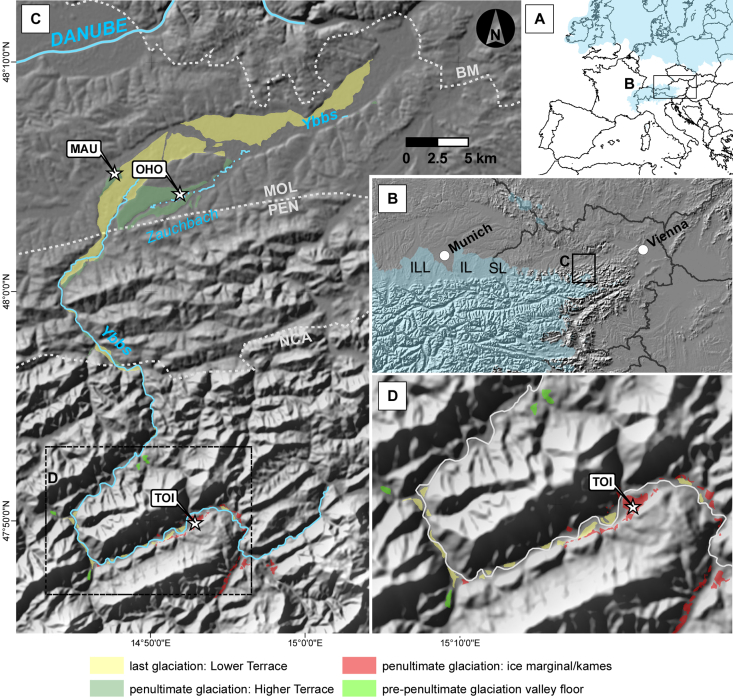
A: Overview of the Alpine and Northern European ice extent during the penultimate glaciation (Ehlers and Gibbard, 2004) B: Ice extent of the Eastern Alps during the penultimate glaciation characterized by piedmont glaciers extending into the Swiss, German and Austrian NAF. Glacial cover of the easternmost part was not as extensive and glaciers often terminated within the mountainous area (e.g. Ybbs valley). ILL … Isar-Loisach glacier lobe, IL … Inn glacier lobe, SL … Salzach glacier lobe C: Study area. Quaternary units are redrawn according to Ruttner and Schnabel (1988), Krenmayr et al. (2006) and Untersweg et al. (2012). Dashed lines represent borders of major geological units (NCA = Northern Calcareous Alps of the Austro-Alpine mega unit, PEN = Penninic and Helvetic Units, MOL = Tertiary molasse, BM = Bohemian Massive). Investigate sites indicated by stars, MAU = Mauer, OHO = Oberhömbach, TOI = Tonibauer/Hochau. D: Detailed view of the inner alpine surroundings of sampling site TOI. Dashed rectangle in figure C indicates the position of the close up.

In this study we investigated the optically stimulated luminescence (OSL) and infrared stimulated luminescence (IRSL) properties of glaciofluvial quartz (Q) and potassium rich feldspar (KFs) deposited during the penultimate alpine glaciation along the eastern alpine Ybbs River (Fig. 1C). For the first time luminescence ages from glaciofluvial sediments attributed to the penultimate glaciation from the eastern part of the alpine realm are presented.

### 1.1 Penultimate glaciation

Depending on regional lithostratigraphy, the penultimate glaciation is termed the late Saalian glaciation in northern Europe (Ehlers et al., 2011b), Illinosian glaciation in North America (Curry et al., 2011), Guxiang glaciation in the Qinghai-Tibetan plateau (Shangzhe et al., 2011), and the Riss glaciation in the classic stratigraphic approach in the Alps (Van Husen and Reitner, 2011a) – all attributed to the late Middle Pleistocene (Cohen and Gibbard, 2011). The most common chronostratigraphic interpretation is a time equivalence with Marine Isotope stage (MIS) 6, however, this is only rarely based on numerical dating. An important step towards a time constraint of the penultimate alpine glaciation was presented by Drescher-Schneider (2000), who could place a sedimentary sequence starting with a basal till to MIS 6–3 based on palynological findings. During the largest extent of the penultimate alpine glaciation, ice advanced into the NAF, where it formed piedmont-style glacier lobes in vast areas (Schlüchter, 1986; Ehlers et al., 2011a). At that time, glacier extent gradually decreased from west to east, with the most extensive piedmont glaciations in the foreland of the Swiss Alps (Fiebig, 2011). The type locality of sediments of the Riss glaciation, which is correlated with the penultimate glaciation, is located in the Riss valley near Biberach in Southern Germany (Penck and Brückner, 1909). Here, the deposits of the Rissian Rhine glacier record a total of three individual glacial advances (Older-, Middle- and Younger-Riss; Ellwanger et al., 2011). In connection with these terminal moraines of the Riss, two gravel terrace levels can be found: The “Upper High Terrace” (“Obere Hochterrasse”, Middle Riss) and the “Lower High Terrace” (“Untere Hochterrasse”, Younger Riss), with the “Upper High Terrace” gravels deposited unconformably above gravels and basal till of the Older Riss (Schreiner, 1989). A trichotomy of the Riss moraines is also observed in the Bavarian and Upper Austrian part of the NAF including two distinct terrace levels (Van Husen, 1977; Kohl, 1999; Doppler et al., 2011).

In terms of stratigraphy, the difference between recent approaches in the Rhine glacier area (Ellwanger et al., 2011) and the stratigraphic approach in the Eastern Alps (e.g. Van Husen and Reitner, 2011a) is the timing and quantity of reconstructed glacials. While the German stratigraphic chart (Stratigraphic Table of Germany (2002)) subdivides the Riss into two discrete glacials separated by a full interglacial (Ellwanger et al., 1995; Miara et al., 1996; Rögner, 2008), Ellwanger et al. (2011) propose a new stratigraphy for the Rhine glacier area in which the Riss is attributed to only one glacial and an additional older glacial (Hosskirch) is introduced. However Van Husen and Reitner (2011a) proposed a stratigraphic model for the Eastern Alps which attributes all Riss deposits to one glaciation correlated with MIS 6 defined by oscillations on a stadial–interstadial scale. A recent overview of the regional stratigraphic interpretations was given in EandG Quaternary Science Journal Vol. 60 No. 2–3 and a brief stratigraphic overview is given in Supplementary table S2. During the maximum extent of the penultimate glaciation, the Ybbs valley glacier was one of the easternmost north draining valley glaciers with a connection to the inner-alpine glacier network (Van Husen, 2000) – even though the glacier was bound to the inner-alpine realm it did not extend into the foreland to form a piedmont style glacier lobe. The extent of mountain glaciers in general depends on several factors, most of all changes in temperature and precipitation (Owen et al., 2009). In contrast to the vast piedmont glaciers of the NAF (e.g. Rhine glacier, Salzach glacier) originating in the highest mountain ranges of the Alps, glacier systems within lower lying mountain ranges (e.g. the easternmost Alps) are likely to react earlier and more sensitive to changes in climatic factors. Therefore insights on the timing of glacial and proglacial processes of the eastern Alpine glacier systems may allow a reconstruction of paleoclimatic conditions during the penultimate glaciation.

### 1.2 Recent dating approaches of terrace gravels in the Eastern Alps and its foreland

Stratigraphic correlations strongly based on morphological indicators of terrestrial archives (e.g. foreland terraces) may be erroneous because of asynchronous formation and/or fragmented conservation of such features. Therefore it is of utmost importance to establish reliable numerical chronologies of such terrestrial archives. Few luminescence ages from loess covering High Terrace gravels have been obtained (Rögner et al., 1988; Miara et al., 1996; Rentzel et al., 2009). However, these ages only provide minimums for the formation of the gravel terraces. The same is true for an important age constraint of High Terrace gravels using U/Th dating of cemented gravel (Terhorst et al., 2002). Direct evidence provided by luminescence ages of the terraces in the NAF is very sparse (e.g. Megies, 2006; Klasen, 2008) and only one High Terrace age of the Austrian NAF has been published. The age (Infrared stimulated luminescence, K-feldspar, not corrected for fading) places the formation of the High Terrace formed by Salzach and Inn glacier melt waters around 170 ± 20 ka (Megies, 2006). Previously, the only comprehensive luminescence dating study of glaciofluvial sediments was carried out by Klasen (2008, see also Klasen et al. 2007) in the NAF of Southern Germany. This study identified analytical problems associated with dating sediments from this area (anomalous fading, incomplete bleaching, age underestimation of quartz), and thus a robust chronology of the terraces could not be established. However, these studies revealed important insights into bleaching processes of quartz and feldspar (Klasen et al., 2006), the fading characteristics of feldspar and the signal composition of quartz OSL (Klasen, 2008). The available luminescence ages of the High Terrace deposits were tentatively correlated with MIS 6 and 8 in southern Germany and Switzerland (Klasen, 2008; Rentzel et al., 2009). With respect to the Austrian NAF, various studies primarily focus on the chronostratigraphy of loess sequences (Preusser and Fiebig, 2011; Terhorst et al., 2011; Thiel et al., 2011a, 2011c; Van Husen and Reitner, 2011b).

In fine-grain luminescence dating approaches (usually using the 4–11 μm grain size fraction) for fluvial and glaciofluvial samples it may be problematic to identify incomplete bleaching (Schielein and Lomax, 2013) because of signal averaging effects (Rhodes, 2007). Also, better bleaching conditions are reported for coarse grained samples (Olley et al., 1998; Vandenberghe et al., 2007; Rittenour, 2008). To maximize the chance of dating well bleached grains and minimizing the effects of signal averaging, we investigated the luminescence signals from small aliquots of coarse grained quartz and feldspar (100–200 μm). Apart from putting a numerical luminescence time constraint on the sedimentary processes of the penultimate glaciation, we explored the luminescence properties of these sediments to assess their suitability for luminescence dating purposes.

## 2 Selected sites and geological setting

The petrographic composition of the catchment area is predominantly made up of carbonate rocks (limestone and dolostone) with only minor input of siliciclastic material (mostly sandstones). Crystalline rocks are completely absent in the River Ybbs catchment. Deposits of the alpine glaciations are characterized by extensive gravel accumulations that form gravel terraces in the foreland, deposited in braided river environments. In the case of the foreland terraces of the penultimate glaciation in the Ybbs valley, a connection to a terminal moraine system (c.f. glacial series) is not given, as terminal moraines of this time are morphologically indiscernible and not exposed. This makes independent numerical chronologies even more important to establish reliable stratigraphic models of such deposits. The sedimentary environment is characterized by heavily gravel-dominated glaciofluvial deposits, with uniformly sorted sandy layers only occurring very scarcely. Caused by the high hydrodynamic depositional setting that also creates erosive unconformities, mostly lenticular sand bodies can be found. These lenses often hardly meet the required sedimentary layer thickness (∼30–50 cm, Preusser et al., 2008) that is desired to guarantee a homogeneous radiation field for a luminescence sample. Proximal deposits in this area tend to be formed in a braided river environment dominated by gravel bar deposits. As Thrasher et al. (2009) pointed out, bleaching probability for sand-sized grains in this kind of environment are relatively low. Increased probability for bleaching may exist in shallow, calm water for bar-top and back-bar sand deposits. Field investigations revealed only three accessible sites (one inner alpine, two located in the NAF) attributed to the penultimate glaciation (Fischer, 1962, 1963, 1996; Nagl, 1968, 1970; Untersweg et al., 2012) which meet the prerequisites for OSL dating as described beforehand. These sites will be described in the following sections using lithofacies codes c.f. Miall (2006) and Thrasher et al. (2009).

### 2.1 Inner alpine site: Hochau

**Fig. 2:**
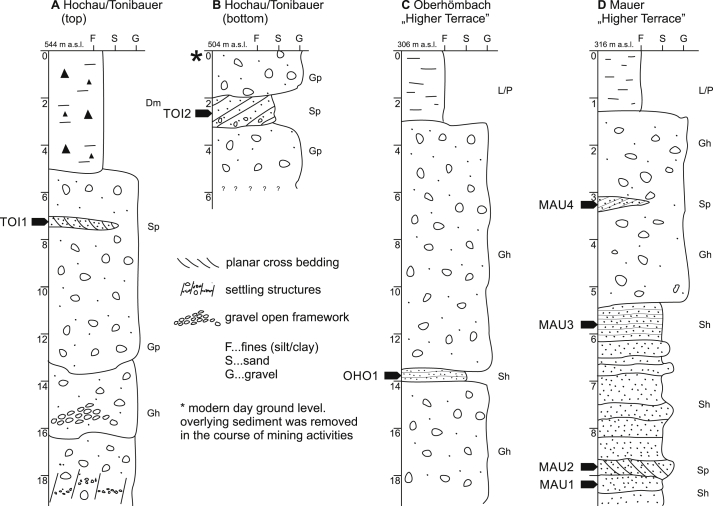
Lithological sections of the study sites. Sampling spots with sample codes are indicated with bold arrows. Facies codes cf. Miall (2006) and Benn and Evans (1998). Sh … horizontal laminated sand, Sp … planar cross-bedded sand, Sl … low angle (<15°) cross-bedded sands; Gp … planar cross-bedded gravel, Gh … clast supported, horizontally, poorly stratified gravel, P/L … fine grained paleosol/loess loam sequence, Dmm … massive diamicton.

**Fig. 3:**
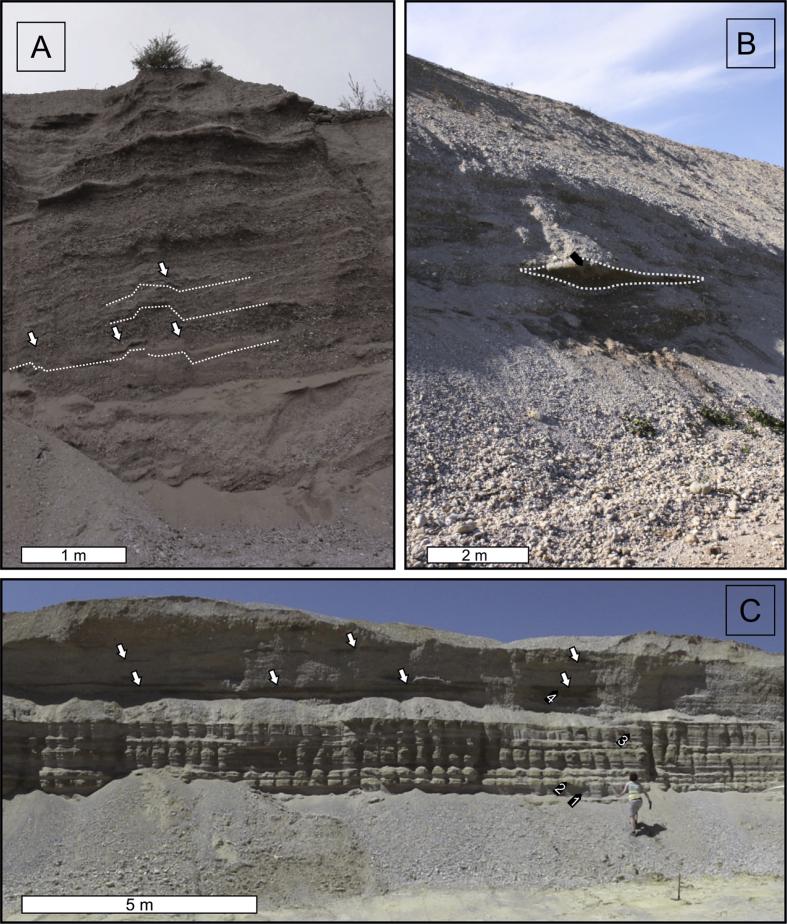
Photographs of the investigated glaciofluvial deposits. A: Inner alpine ice marginal glaciofluvial gravel deposit at Hochau with sedimentary settling structures caused by melting dead ice (white arrows and white dotted lines). B: Horizontally stratified coarse gravel deposit at the Oberhömbach gravel terrace. An intercalated sand lens (dotted line) was sampled for OSL measurements (OHO1, black arrow). C: Mauer sampling site. Bottom: continuous horizontally bedded sand layers. Top: Horizontally stratified coarse gravels with small interbedded sand lenses (white arrows). The bottom part of the section indicates a relatively quiet depositional environment with well sorted and stratified sediments compared to the overlying unsorted gravelly sediments which indicate an environment dominated by strong hydrodynamics. Four samples were taken from this sedimentary succession (black arrows, numbers correspond to sample name, i.e. 1 → MAU1).

The site “Hochau” (UTM 33N E491021 N5297650) is located within Mesozoic units of the Northern Calcareous Alps in the alpine part of the Ybbs valley (Fig. 1). The exposed Quaternary sediment succession exposes one discernable thick gravel body with increasing irregular surface morphology and gradually decreasing thickness towards the valley floor. The unit mainly consists of horizontally bedded, grain supported, coarse gravel and cobble in a coarse sandy matrix (Fig. 2A and B) of mostly carbonate origin. Discordantly cut sand lenses and discontinuous sand layers are scarce and irregularly scattered within the outcrop. Two samples (TOI1, TOI2) were taken from two isolated coarse sand lenses with planar cross stratification (Sp), interpreted as proximal bar-growth deposits. Pure, coarse grained gravel open-frameworks are a common sedimentary feature. This grain size variability indicates a hydrologically highly variable depositional environment. Sediment subsidence structures (e.g. extensional faults; see Fig. 2A lower part and Fig. 3A) are common and interpreted to be caused by post-sedimentary meltout of buried dead ice, indicating deposition of the sediments close to the former ice margin (kame terrace). On top of the gravel deposits lies a 5 m thick cover that consists of a deeply weathered, unsorted diamicton that was previously interpreted as solifluction sediment by Untersweg et al. (2012).

### 2.2 Sites of the alpine foreland: Oberhömbach and Mauer

The two foreland sites are located in the Upper Ybbs valley, 20–25 km to the south-west of today's Ybbs River confluence with the Danube. Both sites are correlated to the High Terrace, a stadial deposit of the penultimate glaciation (Fischer, 1963, 1996; Schnabel, 2002; Krenmayr et al., 2006). Fischer (1996) found a change in drainage direction after deposition of the High Terrace sediments when the Ybbs abandoned a side channel (Fig. 1, Zauchbach) and the discharge shifted more or less towards the modern day river course. At the outcrop of Oberhömbach (Fig. 1, OHO, UTM 33N E489893 N5324284), sediments of the abandoned channel are exposed. 15.5 m of coarse gravels are disclosed in horizontally to sub-horizontally strata (Fig. 2C), where only one layer consists of medium-sized sand suitable for luminescence sampling (Fig. 3B, OHO1).

Four samples were taken at Mauer (Fig. 3C, MAU1–4, UTM 33N E484591, N5325948) which is located 5 km to the northwest of the Oberhömbach site (Fig. 1). The outcrop is dominated by horizontally bedded, coarse gravel deposits, which are separated from the top lying loess loam and carbonate luvisol by a thin, lime-free weathering horizon attributed to an interglacial period (Fischer, 1996, Fig. 2D). Below this horizon there are prominent, continuous horizontal beds of fine-to medium-grained sands, partly cross laminated. Here, three samples for luminescence analysis (MAU1–3) were taken. Thin seams of fine gravel separate the beds. This mostly horizontally stratified sand sequence (Sh) is interpreted as aggradational deposits of a slow flowing water body with intermitting periods of increased discharge (c.f. Miall, 2006). An additional sample was taken from a confined sand lens showing planar cross-bedding (Sp) located within the overlying gravels (MAU4).

### 2.3 Ybbs valley during the penultimate glaciation

**Fig. 4:**
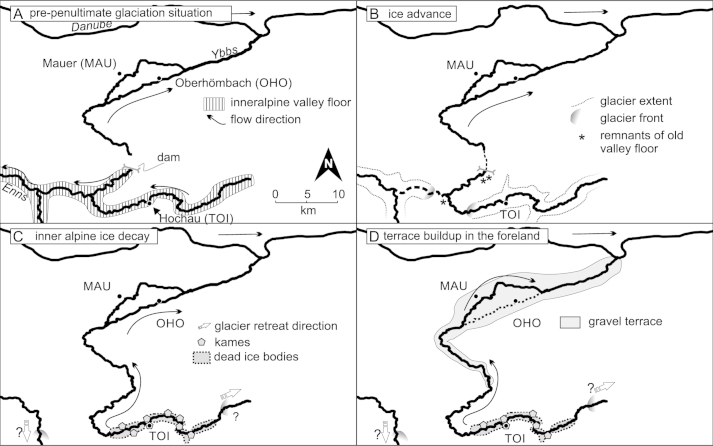
Time sketch of Ybbs valley development. A: Situation before the penultimate glaciation: The Ybbs was flowing to the east which is documented by sedimentary evidence of Ybbs valley provenience in the Enns valley. B: Ice advance during the penultimate glaciation. The connection to the east was blocked by an advancing Enns glacier, damming up a lake which drained northward. C: During the deglaciation phase the glacier melted and disintegrated into several individual dead ice bodies. Sediment was deposited next to and onto these glacier remnants (TOI). D: Quasi-synchronous onset of the deposition of melt water sediments due to massive supply of loose sediment in the foreland of the retreating Ybbs glacier.

Pleistocene sedimentary remnants preceding the penultimate glaciation are scarce in the Ybbs valley. Isolated occurrences of elevated coarse gravel sediments indicate that the pre-penultimate glaciation valley floor could have been considerably higher (Fig. 4A; Untersweg et al., 2012). These deposits were first thought to be in genetic relation to the Hochau sediments as glaciofluvial remnants of an earlier stadial during the penultimate glaciation (Nagl, 1968, 1970) but they were later reinterpreted as possible remnants of an even older valley floor (Untersweg et al., 2012). Sedimentary paleocurrent indicators, base slope, and lithology of these gravels are interpreted as indicators of a westward discharge directed from the Ybbs valley into the Enns valley (Nagl, 1970; Untersweg et al., 2012). No terminal moraines in the foreland from glaciations older than the penultimate glaciation have been found in the Ybbs glacier area (van Husen, 1981) but supposedly older glacial erratics can be found (Ruttner and Schnabel, 1988). During the ice advance of the penultimate glaciation, a sidearm of the Enns glacier blocked the discharge to the west and forced the Ybbs to change its flow direction northward (Nagl, 1970) towards the Danube, much like the modern day river course (Fig. 4B). There are no terminal moraines that mark the maximum extent of the penultimate glaciation in the Ybbs valley. This might be owed to the very narrow valley morphology where sediment relics are only spared from erosion if they are located in sheltered positions (e.g. Hochau). Nagl (1968, 1970) interpreted the Hochau deposits as proximal terrace sediments and attributed the height difference between the surface levels (up to 30 m) to two different glaciation stages, where the higher unit was in direct connection to the ice margin of the Ybbs glacier and the lower unit was the terrace connected to outwash gravels of a younger glaciation which allegedly terminated about 6 km upstream from Hochau. Recent investigations (Untersweg et al., 2012) present a new hypothesis for the valley evolution of the Ybbs valley during the penultimate glaciation: newly found lateral moraine deposits infer that the glacier's most extensive ice advance reached at least 8 km further downstream from Hochau. According to their interpretation, the sediments previously attributed to two different glaciation events by Nagl (1968, 1970; e.g. Hochau) are actually ice marginal remnants (kame terraces) of a long single valley glaciation which were deposited onto and laterally to a glacier decaying into individual dead ice bodies (Fig. 4C). This would result in an irregular surface morphology when sediment covered dead ice is melting. An ice marginal depositional environment is also strongly supported by sedimentary evidence (settling structures, Fig. 3) recorded in the course of this study. Simultaneous deposition of large quantities of loose gravel material would take place in the foreland (sites MAU and OHO; Fig. 4D).

## 3 Equipment and methods

### 3.1 Sampling and sample treatment

Samples for equivalent dose determination were collected by forcing steel tubes into unconsolidated sand layers and sealing the tubes' ends with aluminum foil. Samples for determination of the environmental dose rate were taken from the direct sedimentary surroundings of the luminescence sample (few centimeters radially around the sampling spot). The light exposed ends of the luminescence sample were later discarded in the laboratory under subdued red light conditions. The samples were dried and sieved to extract the grain size fraction of 100–200 μm. The treatment with 10% hydrochloric acid and 15% hydrogen peroxide removed carbonates and organic matter, Sodium oxalate was added to deflocculate clay minerals. Potassium rich feldspar and quartz were concentrated by mineral gravity separation using LST fastfloat (at a density of 2.58 g/cm^3^ and 2.68 g/cm^3^). The quartz concentrate was etched with 40% hydrofluoric acid for 30–40 min to remove the outer layer of the grains affected by alpha radiation and to remove residual plagioclase. The separated quartz was then treated with 15% hydrochloric acid to remove newly formed fluorides. Finally, the etched concentrates were sieved (100–200 μm) to remove smaller grain fragments that resulted from the etching process and may potentially contain feldspar remnants. Feldspar contamination was additionally monitored analytically during the measuring process.

### 3.2 Dosimetry

Environmental dose rates were calculated from measurements of the relevant radionuclides (^40^K, ^232^Th, ^238^U) that contribute to the dose rate using a Canberra high resolution-high purity Germanium gamma detector (40% n-type). ∼1000 g of sample were stored in sealed Marinelli beakers for a minimum of two weeks to ensure secular Rn equilibrium before measurement. The influence of cosmic radiation was calculated according to Prescott and Hutton (1994). Because of mining activity that removed the sedimentary cover, the influence of cosmic radiation in sample TOI2 had to be reconstructed by extrapolating the original burial depth using elevation data of nearby original undisturbed terrain surfaces. Radiation damage caused in non-etched feldspar crystals by α-particles was accounted for by using an assumed mean alpha efficiency (a-value) of 0.07 ± 0.02. This is based on values used by Klasen (2008) for coarse grain feldspar in a similar setting (German NAF). For quartz, the influence of alpha particles was not accounted for, as the rim affected by alpha particle damage of coarse grained quartz was removed by etching (8 ± 2 μm) with 40% HF for 30–40 min. For KFs, a mean internal potassium content of 12.5 ± 0.5% was assumed (cf. Huntley and Baril, 1997). Today's water content was determined by drying the sample at 80 °C which resulted in a measured mean water content of 5 ± 2%. Due to mining activity at all investigated sites these values are regarded as minimum water content because of increased drainage at the gravel pit walls. For this reason, dose rate calculations with a significantly higher water content of 15 ± 10% is assumed to represent the average natural conditions, covering a range from almost dry to almost saturated conditions. This is in the range of water content values reported by other studies dating coarse grain material from similar settings (Klasen et al., 2007; Pawley et al., 2010). Dose rate calculations were performed using the ADELE software (Kulig, 2005) using the dose rate conversion factors by Adamiec and Aitken (1998). Radionuclide concentrations, water content, and effective environmental dose rates are given in Table 1.

**Table 1:** Results of radionuclide analysis and dose rate calculation. Dr_eff = effective dose rate, caused by cosmic and environmental radiation.

Sample	K [%]	±	Th [ppm]	±	U [ppm]	±	Dr_eff [Gy ka^−1^]
TOR1	0.85	0.02	1.25	0.03	4.43	0.13	1.31
APP1	1.04	0.02	9.58	0.26	2.97	0.06	2.86
APP2	1.11	0.02	3.28	0.07	10.54	0.28	3.15
DES1	0.37	0.01	2.00	0.07	1.12	0.03	0.69
DES2	0.27	0.01	1.38	0.05	1.33	0.03	0.63
DES3	1.45	0.03	12.78	0.34	3.53	0.08	3.04
UNT1	0.74	0.02	2.42	0.08	1.27	0.03	0.85
UNT2	0.70	0.00	2.50	0.04	1.09	0.02	1.07
HOR1	0.31	0.01	1.46	0.06	1.40	0.03	0.71
HOR2	0.42	0.01	1.72	0.06	1.23	0.03	0.78
ORO1	0.69	0.02	3.30	0.10	1.54	0.04	1.17
ORO2	0.77	0.02	4.51	0.14	1.90	0.04	1.41
SRN1	0.12	0.00	0.45	0.02	1.93	0.04	0.76
SRN2	0.11	0.00	0.44	0.02	1.93	0.04	0.62
DRF1	0.16	0.00	0.70	0.03	3.06	0.06	0.89
DRF2	1.64	0.04	13.58	0.35	4.10	0.08	3.22
DRF3	0.34	0.01	1.26	0.05	2.36	0.05	0.92
DRF4	0.91	0.02	1.69	0.04	4.09	0.12	1.42
MAU1	0.40	0.02	1.93	0.05	3.81	0.03	1.25
MAU2	0.14	0.03	0.63	0.06	4.03	0.03	1.05
MAU3	0.23	0.02	0.82	0.04	4.38	0.02	1.16
MAU4	0.17	0.03	0.69	0.06	4.55	0.03	1.16
OHO1	0.24	0.02	0.91	0.05	3.77	0.03	1.10
TOI1	0.10	0.03	0.44	0.06	1.97	0.03	0.60
TOI2	0.16	0.02	0.60	0.05	1.98	0.03	0.58

### 3.3 Luminescence measurements

The luminescence was measured in two automated Risø TL/OSL-DA-20 luminescence readers (Bøtter-Jensen et al., 2000, 2003; Thomsen et al., 2006), each equipped with a ^90^Sr/^90^Y beta source delivering a dose rate of ∼0.10 Gy/s and ∼0.13 Gy/s, respectively. Quartz was stimulated by a set of blue LEDs (470 nm, ∼41 mW cm^−2^ @ 100% power) and detected by a photomultiplier tube (PMT) through an ultraviolet transmitting Hoya U340 (7.5 mm) filter. An array of IR emitting diodes was used to stimulate feldspar luminescence which was detected by a PMT using a 410/30 interference filter (2 mm).

Before *D*_e_ measurements, we conducted combined dose recovery preheat plateau tests (DPT) on artificially irradiated (∼155 Gy) 1 mm aliquots for KFs and 4 mm aliquots for Q to assess the reproducibility of a known dose at incremental rising preheat temperatures using the SAR protocol. The aliquots were bleached with the stimulation LEDs of the luminescence reader (blue LEDs for quartz, IR diodes for KFs). Dose recovery preheat plateau tests were performed on MAU2 and MAU4 for quartz andMAU2, MAU3 and MAU4 for KFs using three aliquots per preheat step (KFs: 210–290 °C for 10 s and a 50 °C/225 °C pIRIR protocol; Q: 200–260 °C for 10 s, test dose cutheat 20 °C lower, measured at 125 °C with a standard SAR protocol). Because of low yield in both Q and KFs after the separation process, dose recovery tests for some samples were measured with just one preheat temperature that was inferred from dose recovery preheat plateau tests from MAU2, MAU3,MAU4, and OHO1 (Q 240/220 °C, KFs 250 °C).

For the equivalent dose (*D*_e_) measurements small aliquots of Q and KFs with 1 or 2 mm diameter (approximately 60 grains and 150 grains respectively) were fixed with silicone spray onto 9.7 mm steel discs. We used standard single-aliquot regenerative-dose (SAR) protocols (Wintle and Murray, 2006) for dose determination of OSL of quartz at a stimulation temperature of 125 °C for 40 s. Feldspar post-IR-IR (pIRIR225) doses were determined by an elevated temperature IR stimulation (225 °C for 100 s) following Buylaert et al. (2009). The IR_50_ signal at a stimulation temperature of 50 °C for 100 s of this protocol was also used for *D*_e_ determination (IR50). Signal integrals for quartz OSL were chosen following Cunningham and Wallinga (2010) who use early background subtraction (0–0.4 s signal integral, 0.4–1.4 s background integral). For feldspar IRSL, the first 2 s of the luminescence signal were used and the average background was derived from the last 20 s of each measurement.

The bleaching characteristics of the natural IR50 and pIRIR225 signals were investigated by exposing triple sets of natural 6 mm KFs aliquots of sample MAU4 to direct sunlight over three days of fair weather conditions in July 2013 in Vienna (Austria) with variable exposure times. Aliquots were exposed in following steps: 10 s, 30 s, 1 min, 2 min, 5 min, 15 min, 30 min, 1 h, 2 h, 3 h, 5 h, 1 day/night cycle, 2 day/night cycles, and 3 day/night cycles. Residual doses were determined using the pIRIR-protocol that was used for ED determination (see above). Mean global solar radiation for the experiment site in July is ∼166 kWh/m^2^ (ZAMG, 2002). Bleaching properties for quartz were not investigated, as the OSL signal of quartz resets much faster than IR50 and pIRIR225 signals (Murray et al., 2012; Starnberger et al., 2013).

Additionally, fading tests were carried out on twelve 6 mm aliquots of coarse grained potassium rich feldspars for one sample per site (MAU4, OHO1, TOI1) following the approach described by Auclair et al. (2003). Storage times after irradiation ranged between 10 min and 29 h.

Unfortunately, some quartz samples from the NAF show an unfavorable OSL signal behavior for dating purposes (Klasen, 2008) because their relative medium component contribution in the regenerated signal is higher compared to the natural signal. Furthermore, a thermal instability of the medium component is observed when the sample is thermally stressed. To evaluate the influence of a potentially thermally unstable medium component, pulse annealing experiments were carried out (cf. Fan et al., 2011) on single aliquots of samples OHO1, TOI1, TOI2 and MAU2 with increasing preheat temperatures (200–400 °C in 20 °C steps, 10 s at each temperature; sensitivity correction: test dose 20 Gy, cut heat 200 °C).

Aliquots passing the following SAR quality criteria were used for Q and KFs in this study:

• Recycling ratio 0.9–1.1
• Max. test dose error 10%
• Max. recuperation 5%
• Signal more than 3 sigma above background

Additionally, three “soft rejection criteria” were used for the selection of quartz OSL signals that we incorporated in the *D*_e_ evaluation of each aliquot. First, the initial part of the natural and regenerated decay curves must show a steep slope to background level, indicating a fast component dominated signal. Secondly, aliquots showing substantial change in decay curve geometry between natural and regenerated signals in the SAR sequence were discarded because of possible change in signal component composition. Finally, all aliquots emitting a measurable IRSL signal response after short beta irradiation (20 Gy) and subsequent preheat must be discarded owing to possible feldspar contamination. Given errors in this study are standard errors of the arithmetic mean (1*σ*) except for calculated central ages (central age model, Galbraith et al., 1999) where the standard errors of the model are provided.

## 4 Luminescence properties

### 4.1 Dose recovery preheat plateaus

**Fig. 5:**
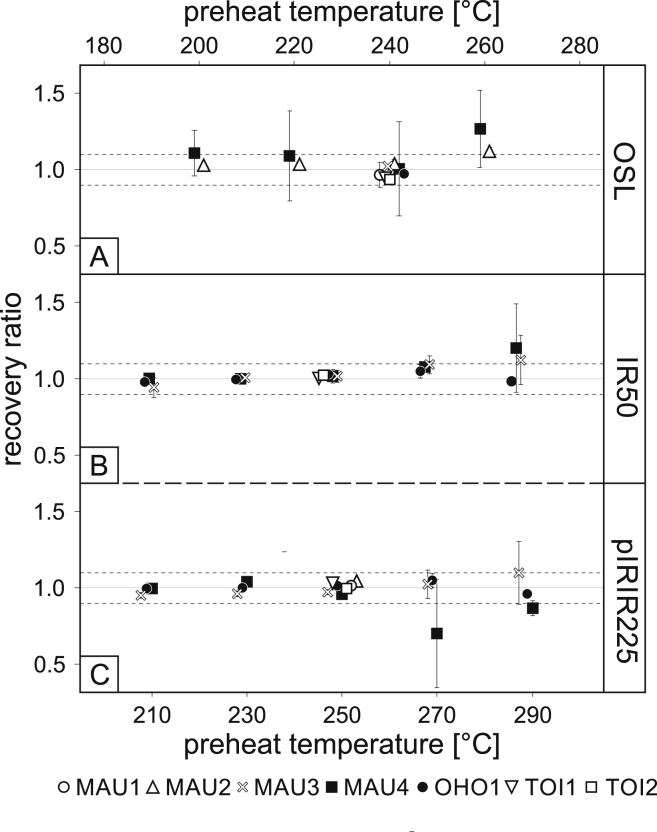
Results of dose recovery experiments (DRT). 3 aliquots per temperature step were measured. Error bars represent 1*σ* standard error. Solid grey line represents unity between given dose and recovered dose. Dashed grey lines represent ±10% of unity, which is the maximum deviation from unity allowed in this study.

The recovered and given doses from all feldspar samples show very good accordance from 210 to 270 °C preheat temperature (Fig. 5B and C). Above 270 °C, the recovered doses for MAU1 and MAU2 deviate more than 10% of the given dose and imply that sensitivity changes in the first measurement cycle are not appropriately corrected for at these temperatures. This result also implies that the high temperature pIRIR_290_ protocol (e.g. Thiel et al., 2011b) cannot be applied to these samples. In summary, good correlation between given and recovered dose was found at a preheat temperature of 250 °C (Fig. 5B and C) which was used for all KFs samples in this study.

The dose recovery tests for quartz were carried out on 6 mm aliquots. Best dose recovery ratios were found for preheat temperatures of 240 °C (Fig. 5A). The dose recovery tests for both mineral types did yield acceptable results (at 250 °C for KFs, 240 °C for Q) in terms of recycling ratio (<10%) and signal recuperation (<5%).

### 4.2 Dose saturation properties

**Fig. 6:**
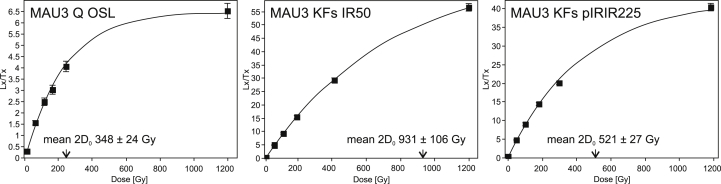
Representative dose response curves for Q OSL, KFs IR50 and KFs pIRIR225 of sample MAU4. Arrows indicate the mean 2D0 value for the respective signal.

Three natural aliquots of each sample were exposed to a high dose (1200 Gy) during the course of a SAR sequence to assess the saturation behavior of the individual signals. By fitting a single saturating exponential growth curve to the regenerated points, the 2*D*_0_ value could be deduced (Fig. 6). 2*D*_0_ values define the upper confidence level, where De's are still ∼15% below laboratory saturation (Wintle and Murray, 2006). Saturation thresholds for all samples in this study were determined using the 2*D*_0_ approach with a high laboratory dose (results given in Table 2). While the OSL signal was in saturation after a 1200 Gy irradiation, the IR50 signal of KFs still did show potential growth (Fig. 6). The pIRIR225 signal did show signs of saturation at 1200 Gy which is also expressed in considerably smaller 2D0 values compared to those of the IR50 signal. In any case, equivalent doses used for age determination were well below the 2*D*_0_ thresholds.

**Table 2:** Summary of OSL and IRSL data. The reliability of ages is based on methodological implications (e.g. age overlap of different signals) intra-section stratigraphic relations and comparison with ages from the same geologic unit (e.g. comparison MAU and OHO). Fading Corrected IR50 ages are not considered for age interpretation and are only discussed as possible minimum ages.

Sample	Location	Signal	Mineral	*n*_total_^a^	*n*_passed_^b^	g-value^c^	De [Gy]	OD [%]	2D0_mean_ [Gy]^d^	Age [ka]^e^
MAU1	Mauer	OSL	Q	72	10	–	145 ± 18	40%	257 ± 83	116 ± 17
		IR50_corr	KFs	38	34	2.89 ± 0.93	292 ± 58	22%	1234 ± 117	156 ± 31
		pIRIR225	KFs	30	21	–	291 ± 19	24%	566 ± 147	**156 ± 16**
MAU2	Mauer	OSL	Q	94	19	–	146 ± 8	15%	262 ± 56	**140 ± 16**
		IR50_corr	KFs	44	41	2.89 ± 0.93	222 ± 9	21%	1205 ± 242	134 ± 21
		pIRIR225	KFs	29	20	–	282 ± 27	40%	770 ± 133	**170 ± 23**
MAU3	Mauer	OSL	Q	56	20	–	147 ± 11	28%	348 ± 26	**127 ± 14**
		IR50_corr	KFs	55	39	2.89 ± 0.93	306 ± 16.0	28%	931 ± 106	171 ± 27
		pIRIR225	KFs	49	18	–	317.0 ± 28	33%	521 ± 27	178 ± 22
MAU4	Mauer	OSL	Q	60	21	–	149 ± 10	24%	253 ± 92	**127 ± 14**
		IR50_corr	KFs	39	31	2.89 ± 0.93	211 ± 20	27%	1258 ± 158	117 ± 18
		pIRIR225	KFs	29	17	0.95 ± 0.47	247 ± 10	4%	844 ± 79	**137 ± 13**
OHO1	Oberhömbach	OSL	Q	32	20	–	158 ± 6	3%	225 ± 37	**128 ± 14**
		IR50_corr	KFs	33	32	2.98 ± 0.59	337 ± 39	27%	1170 ± 214	218 ± 30
		pIRIR225	KFs	29	20	0.88 ± 0.41	544 ± 24	10%	846 ± 73	325 ± 33
TOI1	Hochau/Tonibauer	OSL	Q	39	20	–	85 ± 2	5%	283 ± 67	**142 ± 15**
		IR50_corr	KFs	34	33	3.27 ± 0.44	151 ± 10	11%	937 ± 109	128 ± 6
		pIRIR225	KFs	20	20	0.80 ± 0.49	178 ± 7	9%	740 ± 69	**150 ± 14**
TOI2	Hochau/Tonibauer	OSL	Q	30	22	–	75 ± 3	7%	194 ± 21	**129 ± 14**
		IR50_corr	KFs	30	28	3.27 ± 0.44	176 ± 31	30%	1066 ± 202	158 ± 20
		pIRIR225	KFs	26	23	–	310 ± 18	23%	828 ± 93	279 ± 26

a Number of aliquots investigated.

b Number of aliquots which passed the quality criteria.

c Representative g-values calculated for one sample per site (MAU4, OHO1, TOI1).

d 2D0 mean values derived from three artificially high dosed aliquots for each sample.

e Error: 1 sigma; ages assumed to be the most reliable are printed in bold.

### 4.3 Residual doses of pIRIR225 and IR_50_ signals

**Fig. 7:**
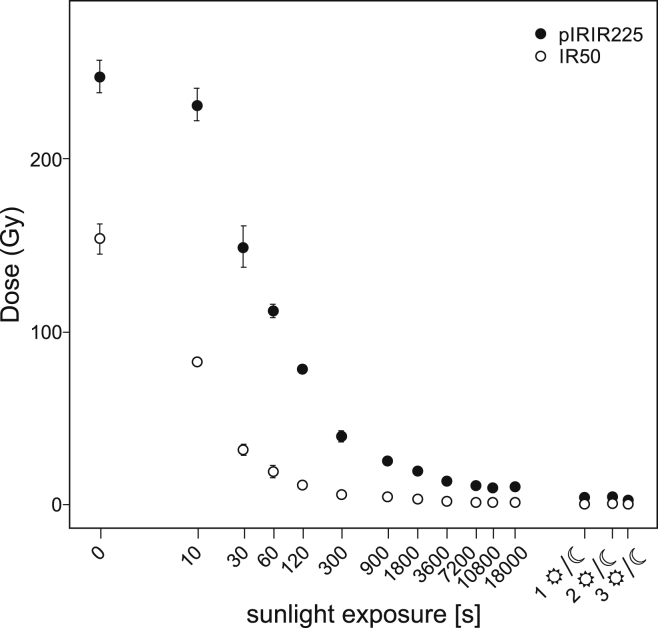
Resetting characteristics for IR50 (open circles) and pIRIR225 (solid circles) signals of KFs sample MAU4 under natural sunlight exposure. Data points represent residual dose recovered after a certain time of exposure to sunlight (error 1*σ* standard error).

The bleaching experiment conducted for the infrared stimulated signals of sample MAU-4 shows that the IR50 signal depletes faster in direct sunlight compared to the pIRIR225 signal (Fig. 7). After 10 s of sunlight exposition IR50 *D*_e_ values dropped to 54 ± 2% of the mean unbleached natural *D*_e_ (154 ± 9 Gy) while the pIRIR225 value only lost 6 ± 4% of its initial dose and started to drop below 50% at exposure times of 60 s and longer. After 2 h of exposure to direct sunlight, IR50 values levelled at a relatively stable residual plateau of <1% (∼1.3 ± 0.2 Gy) of the mean natural IR50 *D*_e._ Similar bleaching characteristics for low temperature IR stimulated feldspar were reported by Godfrey-Smith et al. (1988), and in recent years further investigated by different authors (Buylaert et al., 2012; Li et al., 2014). In contrast, pIRIR225 *D*_e_'s were still around 4.4 ± 0.2% (10.9 ± 0.5 Gy) of the paleodose (247 ± 10 Gy) after two hours of sunlight exposure. Even after exposure times of 3 complete day/night cycles, *D*_e_ values still indicate a slight downward trend, but within *D*_e_ values of 1.0 ± 0.2% of the initial level. Based on these results, samples OHO1 and TOI1 were exposed to direct sunlight under the same perquisites (time, location) as MAU4 for three day/night cycles to estimate the dimension of unbleachable residual doses of the post-IR signal (cf. Buylaert et al., 2011; Reimann et al., 2011; Thiel et al., 2011a,b,c). MAU4, OHO1 and TOI1 show residual doses below 1 Gy for the IR50 signal and below 4 Gy for the pIRIR225 signal. As these small residual doses have a negligible influence on the paleodose of old samples (expected *D*_e_ IR50 > 100 Gy, pIRIR225 > 150 Gy), no residual dose correction was applied.

### 4.4 Fading of KFs signals

**Fig. 8:**
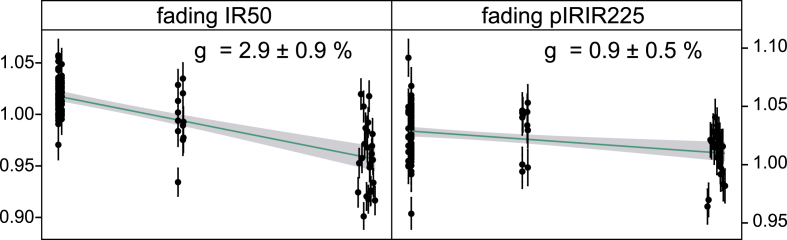
Results of laboratory fading tests for one sample of each investigated site. IR50 and pIRIR225 fading rates are rather uniform (∼3%/decade and ∼0.9%/decade).

Samples investigated for fading (TOI1, OHO1, MAU4; 1 sample per location) show mean fading rates (g-value) for the IR50 signal between 2.9% and 3.2% overlapping within one standard deviation (Fig. 8). Even though most of the measured *D*_e_ values plot on the exponential part rather than the linear part of the growth curve, IR50 ages were corrected using the method of Huntley and Lamothe (2001). There are reports, that age underestimation after fading correction following Huntley and Lamothe (2001) for the exponential part of the dose response curve is indeed significant but not necessarily severe (Auclair et al., 2007) However, age underestimation of the true depositional age cannot be quantified and ruled out completely; therefore all presented fading corrected IR50 ages are interpreted as minimum ages. The fading rates are rather uniform throughout the whole study area and are not expected to change for feldspars from the same sedimentary unit. Therefore the calculated g-values for TOI1 and MAU4 were used for remaining samples of the same site (TOI2 and MAU1–3). Results of the correction are discussed in Section 5.

The observed pIRIR225 fading rates were considerably lower compared to IR50 signals with g-values averaging at 0.9%. The different fading rates of both IR signals in this study are in agreement with previously published studies (Thomsen et al., 2008; Buylaert et al., 2009; Thiel et al., 2011b). Various studies regard such low pIRIR225 values as laboratory artifacts (Buylaert et al., 2011; Thiel et al., 2011b) and hence these are not corrected for here.

### 4.5 Thermal stability of quartz

**Fig. 9:**
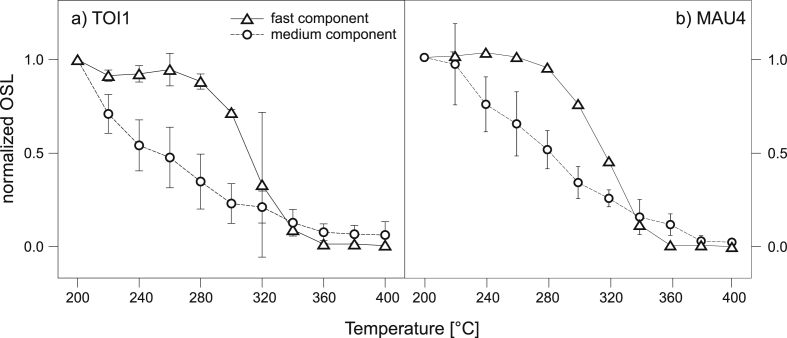
Results of the pulse annealing experiment of sample a) TOI1 and b) MAU4. Datapoints represent the normalized, sensitivity corrected OSL signal with 1 *σ* standard error. The fast component is stable up to a preheat temperature of 280 °C for both samples. In contrast, the medium component has a gradual decreased signal intensity from 220 °C and higher for TOI1 and 240 °C and higher for MAU4.

OSL signal data from pulse annealing experiments was imported in an R software environment and deconvoluted using the “Luminescence” package (Kreutzer et al., 2012) using a function with multiple, overlapping first order exponentials to split the OSL signal into its individual components (Jain et al., 2003). The experiments (Fig. 9) revealed a decrease of the medium component contribution in the regenerated signal with increasing preheat temperatures. The decrease is evident for TOI1 and OHO1 from the first increased preheat temperature step and upward, while the medium component OSL of MAU4 started to decrease significantly at 240 °C and higher. While the bulk signal influence of the medium component for OHO1 and TOI1 is relatively low (around 20% of the initial 0.1 s) it can contribute to almost 40% of the initial signal intensity for the investigated aliquots of sample MAU4. Similar behavior was also observed for sample MAU2. When present, a thermally unstable medium component in the initial part of the signal may lead to dose underestimation due to differential behavior of the natural and regenerated signals (Steffen et al., 2009). To minimize the influence of a thermally unstable medium component in the course of *D*_e_ measurements, quartz OSL signals were carefully selected according to OSL decay rate and were evaluated using an early background approach (Cunningham and Wallinga, 2010). On the other hand, the fast component shows a relatively stable plateau up to temperatures of 280 °C and significantly decreases at temperatures at and above 300 °C for MAU4 and TOI1. OHO1 shows the first significant drop in signal intensity for the fast component at a temperature of 320 °C. In summary, for the sedimentary catchment area we assume that the fast component in our samples is stable up to a temperature level of at least 280 °C.

## 5 Equivalent dose distribution and age calculations

### 5.1 Inner-alpine deposits (TOI)

**Fig. 10:**
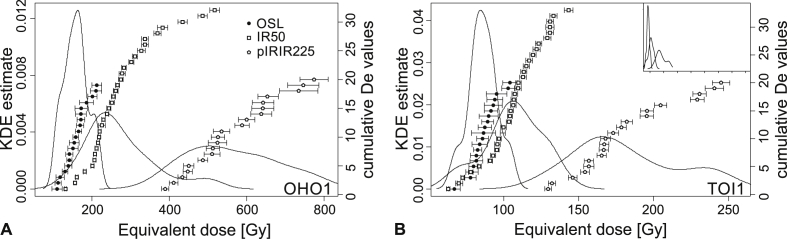
Cumulative dose distribution and kernel density plots of two representative samples for OSL, IR50 and pIRIR225 signals. For dose distribution of other samples see supplement S1. Note the different scales of the *x*-axis in figure A+B. While De's for OHO1 (A) range from 110 Gy to almost 800 Gy, De's of TOI1 (B) range from 70 to around 250 Gy. Inset (right top) shows KDE for TOI1 scaled to *x*-axis of A. Apart from obvious differences of effective dose rate between both sampling sites, also differences in bleaching may account for this discrepancy.

**Fig. 11:**
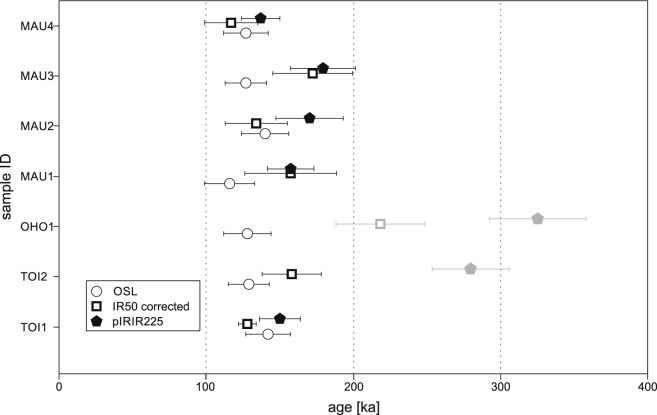
Comparison of CAM ages for OSL, IR50 (corrected and uncorrected) and pIRIR225 signals.

Both quartz samples show a narrow, uniform *D*_e_ distribution (Fig. 10B) even though the gained natural signals mostly intersect the dose response curve in the non-linear part. This is somewhat unexpected, as dose distributions from old samples derived from the non-linear part of the saturating growth curve often result in asymmetric distributions (Murray and Funder, 2003). In contrast, the distribution of IR50 *D*_e_ values is broader. When comparing *D*_e_ values of IR50 we found that sample TOI1 has a symmetrical distribution with values that span a range of ∼100 Gy whereas the range of *D*_e_ values of the same signal is doubled in TOI2 (over 200 Gy). *D*_e_ values are scattered even wider for pIRIR225 signals of both samples; the largest span was found in sample TOI2, where *D*_e_'s covers a range of over 300 Gy. Quasi-Gaussian distribution of all quartz samples indicate that quartz is bleached completely (e.g. Wallinga, 2002). In this case it is appropriate to apply the central age model (CAM, Galbraith et al., 1999) to estimate the accurate paleodose of a set of aliquots. The calculated central doses are 85 ± 2 Gy for TOI1 and 75 ± 3 Gy for TOI2 with low overdispersion of 5% and 7% (Table 2). The resulting ages of 142 ± 15 ka (TOI1) and 129 ± 14 ka (TOI2) overlap within 1 sigma (Fig. 11). Applying the CAM to the IR50 distribution gives ages of 90 ± 8 ka (TOI1) and 95 ± 11 ka (TOI2). When this is corrected for fading the ages increase to 128 ± 6 ka and 158 ± 20 ka respectively (OD_TOI1_IR50_ = 11%, OD_TOI2_IR50_ = 30%). CAM ages for the pIRIR225 *D*_e_ values result in 150 ± 14 ka for TOI1 and 279 ± 26 ka for TOI2 (OD_TOI1_pIRIR225_ = 7%, OD_TOI2_pIRIR225_ = 23%). As the three different signals have different intrinsic bleaching properties, an offset in ages calculated with the same statistical approach is a strong indicator for incomplete bleaching (Murray et al., 2012; Kars et al., 2014). Quartz, fading corrected IR50 and pIRIR225 ages for TOI1 all overlap within 1 sigma standard error. The same can be observed for the OSL and fading corrected IR50 ages of TOI2 but the pIRIR225 age shows a significant offset from the ages gained from the quartz OSL signal. We attribute this behavior to incomplete bleaching of the post-IR signal prior to deposition for sample TOI2. In contrast, TOI1 seems to be well bleached which is supported by overlapping ages derived from all three evaluated signals. Resulting *D*_e_ values and ages are given in Table 2.

### 5.2 Sediments of the alpine foreland (OHO and MAU)

OHO1 *D*_e_ distributions (Fig. 10A) of quartz are similarly narrow shaped like sample TOI2, whereas IR50 and pIRIR225 *D*_e_‘s cover a relatively wide *D*_e_ range (IR50: 132–517 Gy, pIRIR225: 390–774 Gy). Compared to the quartz CAM age of 148 ± 16 ka, the fading corrected IR50 age of 218 ± 30 ka and a pIRIR225 age of 325 ± 33 ka are considerably higher. We interpret these offsets between quartz, IR50 and pIRIR225 ages to be caused by incomplete bleaching. Because quartz OSL signals are bleached more rapidly in nature compared to both investigated IR signals (KFs), the quartz age from OHO1 is regarded to be the most reliable (Fig. 10A). Although differences in calculated ages indicate incomplete resetting of the luminescence signal, overdispersion values are low in particular for pIRIR225 (OD_OHO1_pIRIR225_ = 10%).

Ages of the Mauer site were calculated using the central age model as well. The samples which were taken out of the continuous horizontal sand layers (MAU1–3, Figs. 2 and 3) gave a mean CAM quartz age of 128 ± 12 ka. Of all measured aliquots, only 13% passed the rejection criteria. 64% of aliquots were discarded because of failure to meet set SAR quality criteria (low signal to noise ratio, recycling ratio, etc.) and additionally 23% of measurements were discarded due to suspicious signal behavior (slow signal decay, change in decay curve geometry, feldspar contamination, see 3.4) regardless of passing the SAR error threshold. MAU4 yielded an OSL age of 134 ± 15 ka. All quartz ages for this site overlap within their respective 1 sigma error margins. This also applies for the calculated feldspar pIRIR225 ages, which overlap in a 1 sigma range with central values between 137 and 178 ka (Fig. 11). No significant offsets between pIRIR225 and OSL ages that hint towards incomplete bleaching or influence of a thermally unstable medium component as 2 ages overlap within 1 sigma and all ages overlap within 2 sigma. After correcting IR50 *D*_e_s for fading, the ages agree with pIRIR225 ages within uncertainties but are only regarded as potential minimum sedimentary ages. Resulting ages and equivalent doses are shown in Table 2.

## 6 Discussion

### 6.1 Methodological implications

Even though short transport distances are to be expected for the ice marginal deposits of Hochau (TOI), incomplete resetting does not pose a problem for the quartz OSL signal in both samples. All investigated signals (OSL, IR50, pIRIR225) for TOI1 overlap within 1 sigma. We interpret overlapping OSL and pIRIR ages on the one hand as signs of complete resetting prior to deposition and on the other hand as indication of negligible influence of an eventual thermally unstable component in the OSL signal and athermal fading in the pIRIR signal on the calculated age. The quartz age of TOI2 is consistent with the quartz age of TOI1. Incomplete bleaching of TOI2's pIRIR225 signal seems to be an issue as the age calculation using this signal overestimates the OSL and IR50 ages by ∼100 ky. Even though perquisites were taken to sample deposits with an increased probability of complete resetting (see 2.1 and 2.2), samples from similar depositional environments do not necessarily show the same bleaching condition which once more confirms that bleaching is a heterogenous process (Murray and Olley, 2002). Gaar et al. (2013) found similar discrepancies in bleaching conditions in two samples of one and the same glaciofluvial deposit in the NAF of Switzerland.

Post IR as well as fading corrected IR50 ages from Oberhömbach (OHO) seem to be vastly overestimated when they are compared to luminescence dating results of the nearby Mauer site. In addition, the geomorphological position of the Oberhömbach site and results of previous field studies (Nagl, 1968, 1970; Fischer, 1996) suggest contemporary deposition at the Oberhömbach and Mauer sites. Unbleachable residual doses are ruled out as a reason for pIRIR225 overestimation and incomplete bleaching seems to be the most likely cause. Reports from other parts of the Eastern Alps (Klasen et al., 2007; Klasen, 2008) indicate that partial bleaching is a common problem among this type of sediments. In contrast, in our study incomplete bleaching is mainly a problem (if present) that inherits feldspar signals and quartz seems to be relatively well bleached throughout.

In contrast to the samples from Mauer, the Oberhömbach sample received additional sediment input from a short tributary river (Fig. 1, Zauchbach) that erodes almost exclusively siliciclastic material from the Flysch zone (mainly clay- and sandstones). The distance between the modern day Zauchbach spring and the assumed confluence with the Paleo-Ybbs River is only ∼5 km. This increases the chance of poorly bleached sediment input from a tributary river. Low overdispersions are often regarded as indicators for complete resetting of the natural signal (Olley et al., 2004; Bateman et al., 2008; Neudorf et al., 2012). Although the pIRIR225 age of OHO1 shows low overdispersion values (∼10%), it overestimates the quartz age by ∼200 ka. Wallinga (2002) indicated, that broad but symmetric distributions (thus low overdispersion) also can show incomplete bleaching if the count of poorly bleached grains is high. Therefore overdispersions calculated from the Galbraith et al. (1999) central age model can only be regarded as vague indicators of incomplete bleaching in this study. Unbleachable residual doses are ruled out as a significant contributor to De overestimation of pIRIR signals, as they only represent <1% of the total equivalent dose and therefore can be neglected.

In recent years, evidence has been found that high dosed quartz samples measured by SAR may exhibit systematic age underestimation (Murray et al., 2007; Lowick and Preusser, 2011; Timar-Gabor and Wintle, 2013). The quartz ages presented in this study are unlikely to be underestimated due to high dosage, as all ages from different samples agree within error even though some samples are relatively high dosed (e.g. OHO1: 158 Gy) and others show a relatively low equivalent dose (e.g. TOI2: 75 Gy).

### 6.2 Valley evolution in the light of luminescence ages

**Fig. 12:**
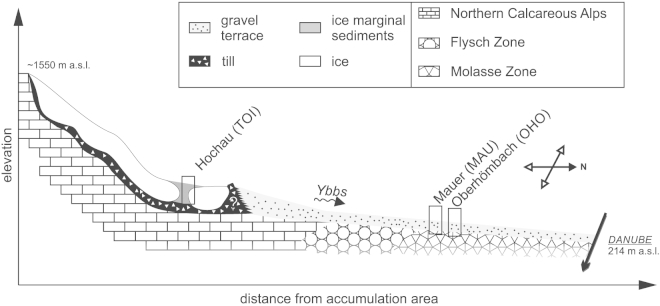
Synoptic lateral profile sketch of the Ybbs valley. Elevations and distances of the illustration are not to scale.

The luminescence ages strongly support the hypothesis of Untersweg et al. (2012) that the sedimentary remnants of the penultimate glaciation indeed did form during a single glaciation event. Calculated quartz OSL ages for the TOI samples (Table 2) indicate that ice decay and associated deposition of the kame terrace happened in closing MIS 6. A synoptic lateral profile sketch is displayed in Fig. 12, showing the genetic relationship of the investigated sites.

U/Th dating on calcite cements of the topmost gravel deposits at Hochau revealed that polyphase cementation took place at 56.8 ± 2 ka and 79.7 ± 2 ka (Untersweg et al., 2012). Calcite cements from delta sediments deposited in front of the Ybbs glacier ∼8 km downstream from the Hochau sediments yielded U/Th cementation ages of 56 ± 2 and 115 ± 2 ka (Untersweg et al., 2012). Such U/Th cementation ages from a glaciofluvial sedimentary environment have to be considered as post-depositional ages and support the luminescence ages presented in this study.

There is weak geomorphological indication (slight altitudinal offset of terrace levels) that the OHO sediments could have been deposited before the MAU terrace sediments (Fischer, 1996). However, chronological differentiation of the depositional age between the supposedly younger Mauer gravel terrace and the older Oberhömbach gravel terrace is not possible by means of luminescence dating because of overlapping ages. pIRIR225 and quartz OSL ages from the Mauer site all indicate deposition in relatively short succession, with no significant age hiatus in between. Calculated ages indicate that the accumulation of the gravel terraces in the alpine foreland of the Ybbs valley most likely happened during middle to late MIS 6 stage and that the terraces of Mauer and Oberhömbach can be attributed to the same depositional unit, showing a simultaneous or at most a slightly temporally delimited sedimentation (Table 2, Fig. 4D). As the gravel terrace of Oberhömbach was not incised by later Ybbs River activity (e.g. during the last glacial cycle), the change of river course must have happened after the deposition of the High Terrace sediments.

### 6.3 The “Riss” glaciation

According to Nagl's (1968, 1970) model, TOI1 is supposed to correspond to the older Riss and TOI2 to the younger Riss. However, the luminescence ages of both samples in combination with sedimentary evidence of deposition onto ice, strongly favor the concept of a single kame terrace accumulation during a final retreat phase of the penultimate glaciation. If there had been a two-phase Riss glaciation, with an older, more extensive glacier advance (Nagl, 1970), the blocking of drainage to the west by the Enns valley glacier would have caused the Ybbs to redirect north and therefore also deposit its coarse bedload to the north in two phases. However, as only a single High Terrace level is present in the Ybbs catchment area, this scenario seems unlikely. Terrace accumulation ages (Table 2) of the Mauer and Oberhömbach terrace also indicate deposition towards the end of MIS 6 when large amounts of loose sediment was available in the forefront of the retreating glacier.

**Fig. 13:**
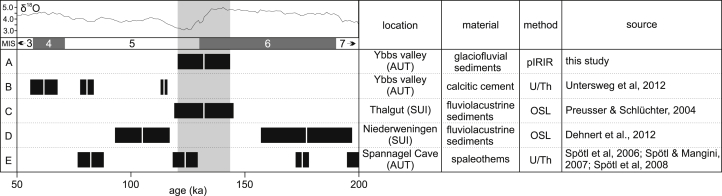
OSL ages from the Ybbs valley in comparison with numerically dated terrestrial records of the greater alpine region. Benthic δ^18^O stack and marine isotope stages (Lisiecki and Raymo, 2005) are shown on top of the illustration for orientation purposes. A: Mean OSL age of the glaciofluvial deposits of the Ybbs valley (MAU1–4, OHO1, TOI1–2). B: U/Th ages of calcite cement generations of glaciofluvial deposits of the Ybbs valley. C: Mean OSL ages of fluviolacustrine sediments of the Thalgut site (THG1–3) in Switzerland. The authors interpret these sediments to represent the transition between the penultimate glaciation and the last interglacial (Preusser and Schlüchter, 2004). D: Mean OSL ages of lacustrine samples of the Niederweningen drill core NW09 (Dehnert et al., 2012). The dated sediments indicate a cold climate, proximal lacustrine environment with two ice advances between 185 ka and 140 ka. E: U/Th dated speleothem growth phases of the Eastern Alpine Spannagel Cave (Austria, Spötl and Mangini, 2007). No growth was recorded between ∼130 and ∼170 ka, indicating conditions within the cave which prevented speleothem growth (e.g. temperatures below freezing).

The OSL ages presented in this study coincide with the Beringen glaciation of the Western Alps which was dated to 180–130 ka (Preusser and Schlüchter, 2004; Dehnert et al., 2010; Preusser et al., 2011) and later subdivided into two separate ice advances at ∼185 ka and ∼140 ka (Dehnert et al., 2012). Fig. 13 shows a comparison of numerical dating results discussed in this section. Luminescence ages obtained in this study do not point towards a two-phased Riss glaciation in the Ybbs valley, but sediments deposited during a hypothetical first glacier advance could have been eroded or be so far unrecognized as such. A transitional sequence from high energy glaciofluvial gravels of the penultimate glaciation to low energy lacustrine fine sedimentation attributed to the last interglacial in the Western Alps is documented in the site of Thalgut (Schlüchter, 1989). OSL dates of the sandy transitional zone (Preusser and Schlüchter, 2004) revealed a deposition at c. 130 ka, which correlates well with the dated ice decay in the Ybbs valley, probably indicating that climatic amelioration happened simultaneously in the Western and Eastern Alps. Definitive evidence for the presence of full interglacial climatic conditions in the Eastern Alps is given in the U/Th-dated speleothem records of the high-lying Spannagel Cave (Austria) which indicate ice free conditions around 125 ka (Spötl and Mangini, 2006; Spötl et al., 2006). As ice in the Ybbs valley was still present around 135 ka near Hochau (TOI1–2) a rapid transition to interglacial conditions is very likely (i.e. Termination II, Drysdale et al., 2009). In contrast, U/Th ages of calcite cements of glaciofluvial gravels of the Ybbs Valley only allow precise determination of the formation of the cement, but only permit minimum estimates of the timing of the initial deposition process.

Compared to the northern European glaciation, the timing of the ice decay of the Ybbs glacier chronologically correlates with the final stage of the Saalian glaciation in northern Germany (“Warthe stage”, Litt et al., 2007) where quartz derived from glaciofluvial sandur sediments was dated by luminescence methods between 150 and 130 ka (Lüthgens et al., 2010) and OSL ages of Danish fluvial and glaciofluvial sediments deposited above a till layer indicate that deglaciation during the Warthe stage started as early as ∼165 ka (Houmark-Nielsen, 2007).

Although the luminescence ages presented in this study are able to place the Riss glaciation in the middle to late MIS 6, the ages are still afflicted with uncertainties covering many thousands of years. While luminescence ages permit detailed comparison of sedimentary/climatic archives on a millennial scale for the last glaciation, the results of old deposits (>120 ka) are inherently limited to a lower resolution. Nonetheless, luminescence dating of old sediments can provide valuable information about the timing of depositional phases, even more when combined and compared with different dating approaches (e.g. U/Th dating).

## 7 Conclusions

Seven samples from glaciofluvial deposits associated with the penultimate glaciation in the Alps and NAF of Austria were dated by luminescence methods. We investigated three different luminescence signals of quartz and feldspar (OSL, IR50, pIRIR225) to identify issues of incomplete resetting prior to deposition. Based on these results, a reliable luminescence chronology, based on quartz OSL and KFs pIRIR225 ages was established for deposits associated with the penultimate glaciation of the Ybbs valley. This luminescence chronology is supported by field evidence and U/Th ages (Untersweg et al., 2012).

In this study we could confirm for the first time based on multiple evidence of well-defined proglacial sediments that the penultimate glaciation (Riss) of the Eastern Alps is time-equivalent to MIS 6. The timing of the deglaciation phase of the penultimate alpine glaciation could be narrowed down to late MIS 6 by dating ice marginal sediments deposited on, and next to a glacier decaying into individual dead ice bodies. This process coincides with the depositional ages of distinct gravel terraces in the NAF of the Ybbs valley. In comparison with previous studies of the Western Alps (e.g. Preusser and Schlüchter, 2004) we conclude that the deglaciation of the Western and Eastern Alps was a contemporary process. However, to corroborate these findings, our first results from the Ybbs catchment area need to be extended by further studies on a larger regional scale.

## Appendix A Supplementary data

Supplementary Fig. S1 Dose distribution plots of samples MAU1, MAU2, MAU3, MAU4, TOI2.

Supplementary Fig. S2 Schematic chronostratigraphic overview of the penultimate alpine glaciation in several stratigraphic models.
