# Supplementary material for: Luminescence dating of glaciofluvial deposits linked to the penultimate glaciation in the Eastern Alps
Source: Quat Int. 2015 Jan 30;357:110–24. doi: 10.1016/j.quaint.2014.10.013 (PMC4394144; doi:10.1016/j.quaint.2014.10.013)

Plot of Dose distribution data MAU1

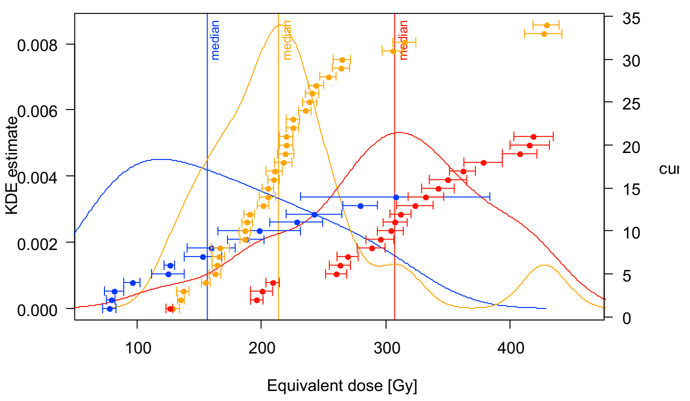

Plot of Dose distribution data MAU2

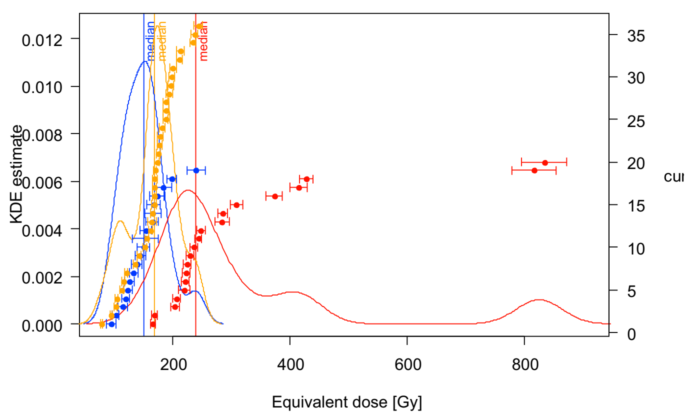

Plot of Dose distribution data MAU3

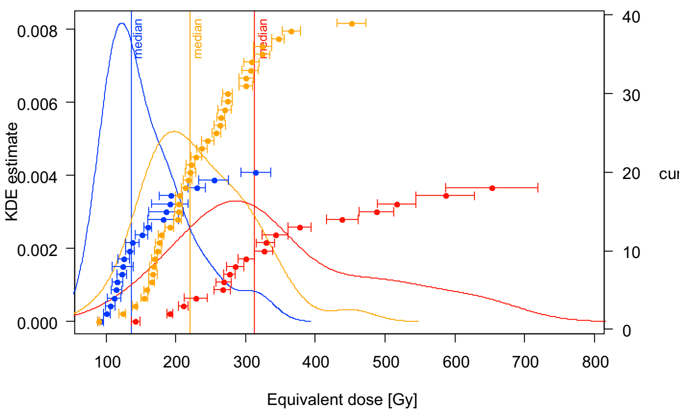

Plot of Dose distribution data MAU4

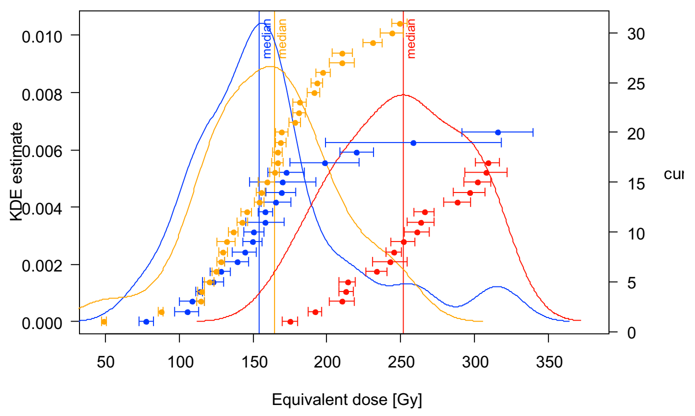

Plot of Dose distribution data TOI2

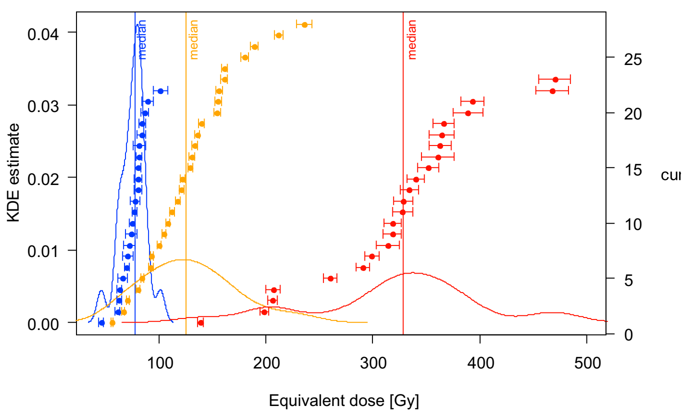

Supplement: Supplementary Fig. S1 — Dose distribution plots of samples MAU1, MAU2, MAU3, MAU4, TOI2. [file mmc1.pdf]
