# Supplementary material for: Luminescence dating of glaciofluvial deposits linked to the penultimate glaciation in the Eastern Alps
Source: Quat Int. 2015 Jan 30;357:110–24. doi: 10.1016/j.quaint.2014.10.013 (PMC4394144; doi:10.1016/j.quaint.2014.10.013)
Supplement: Supplementary Fig. S2 — Schematic chronostratigraphic overview of the penultimate alpine glaciation in several stratigraphic models. [file mmc2.pdf]

<table>
<tr><th></th><th>Switzerland
Preusser et al., 2011</th><th>Baden-Wuertemberg
Ellwanger et al., 2011</th><th colspan="2">Bavaria
Doppler et al., 2011</th><th>Austria
van Husen & Reitner, 2011</th><th colspan="2">Ybbs valley
c.f. Nagel, 1970</th></tr>
<tr><td rowspan="8">Middle Pleistocene</td><td colspan="2">Eem</td><td colspan="5">Riss/Würm Interglacial</td></tr>
<tr><td rowspan="2">Beringen glaciation</td><td rowspan="6">Riss</td><td rowspan="6">Riss-Complex</td><td>Jungriss</td><td rowspan="6">Riss</td><td rowspan="6">Riss</td><td rowspan="3">Spätriss</td></tr>
<tr><td rowspan="3">Mittelriss</td></tr>
<tr><td rowspan="3">Hagenholz glaciation</td></tr>
<tr><td rowspan="3">Hochriss</td></tr>
<tr><td rowspan="2">Altriss</td></tr>
<tr><td>Habsburg glaciation</td></tr>
<tr><td>Thalgut interglacial</td><td>Holstein interglacial</td><td colspan="5">Mindel/Riss Interglacial</td></tr>
</table>
